# Healthcare interventions to aid patient self‐management of lower limb wounds: A systematic scoping review

**DOI:** 10.1111/iwj.13969

**Published:** 2022-10-21

**Authors:** Layla Bolton Saghdaoui, Smaragda Lampridou, Simona Racaru, Alun Huw Davies, Mary Wells

**Affiliations:** ^1^ Imperial College Healthcare NHS Trust & Imperial College London UK; ^2^ Preventative Cardiology, Imperial College Healthcare NHS Trust & Imperial College London UK

**Keywords:** chronic wound, health behaviours, lower limb, self‐management, self‐care

## Abstract

Chronic lower limb wounds can be described as having the inability to progress through stages of wound healing. Although 80% of lower limb wounds develop as a result of venous insufficiency, other causes include arterial disease and diabetes. In addition to the sustained impact on quality of life, the chronicity of lower limb wounds presents a significant financial burden to healthcare systems. Self‐management is a fundamental aspect of the long‐term management of chronic illness and its relevance has intensified since the start of the global pandemic. The objective of this systematic scoping review was to define what the self‐management of a lower limb wound entails and explore the interventions available to support patients to self‐manage. A total of seven articles were evaluated. There was limited consensus regarding the definition and components of self‐management in this area. Interventions involved patients participating in additional exercise, wound care, and lifestyle behaviours such as limb elevation and skin care. Only two studies applied theory and only one evaluated participant acceptability of interventions, making it difficult to assess the feasibility of implementation. Although the evidence reviewed provides some insight into the self‐management of a lower limb wound, theoretically‐guided research is needed in this area.

## 1 INTRODUCTION

There is limited consensus regarding a recognised definition for chronic wounds however they can be described as wounds which are unable to progress through the orderly and timely stages of wound healing. Chronic lower limb wounds include, but are not limited to, those caused by venous, arterial, and diabetic ulceration, with venous ulceration accounting for 80% of all ulcers^1^, ^2^ The negative impact of lower limb wounds on the quality of life of patients is well documented and affects all domains of daily living.^3^ Chronic lower limb wounds also present a significant financial burden accounting for an estimated £3.1 billion of NHS funding per annum.^4^

Self‐management is fundamental to the long‐term management of chronic illness and has been shown to improve clinical outcomes, quality of life and result in fewer hospitalizations.^5^ The burden of chronic conditions as a whole has led to the NHS long‐term plan making a commitment to improve personalised care through the development of supportive self‐management.^6^ This commitment has been pushed to the forefront of healthcare delivery due to the covid‐19 pandemic causing many patients difficulty in accessing healthcare services.^7^

Self‐management of a chronic condition can be simply defined as a person's active participation in their treatment.^8^ Participation can be broken down into three key components; medical management, behavioural management, and emotional management.^9^ In the case of lower limb wounds, medical management could include the following: wound cleansing; primary dressing application; and compression therapy. Types of behavioural management may include adherence to prescribed treatment regimes or lifestyle adaptations such as the incorporation of exercise into the daily routine. Living with a lower limb wound can be associated with loneliness and isolation, therefore emotional management is also an important area of consideration.

To address the urgent need for adaptations to healthcare delivery in response to the COVID‐19 pandemic, the UK's National Wound Care Strategy Programme published guidance regarding how healthcare providers can best support patients with lower limb wounds to adopt self‐management behaviours. This included providing patients with the appropriate support via telemedicine, ensuring they are aware of concerns and warning signs in addition to offering compression hosiery or wraps where appropriate.^7^

Although the benefits of the continued use of compression hosiery, regular exercise, and limb elevation have been acknowledged, in practice patients often face significant barriers.^10^ Adherence to lifestyle and self‐management strategies is often low, as patients report conflicting advice and have difficulty tolerating and applying hosiery.^10^ Furthermore, wound therapy can be daunting for patients, and often the decision to self‐manage stems from poor experiences of healthcare services.^11^ Many opt to self‐manage with dressings and wound cleansing as it allows for greater independence however, very few are totally self‐sufficient. A survey of 100 patients living with a chronic wound found that, in addition to the support of healthcare professionals, over 80% of respondents were assisted with wound care by a domestic partner, relatives, friends or neighbours. Although 91% of this sample of patients carried out wound care independently, only 6% reported receiving prior education or supervision at the start of self‐treatment.^12^ Qualitative studies also reveal that many patients observe wound therapy techniques used by hospital or home‐care staff and attempt to replicate these themselves, often with no initial supervision.^13^ As a result it is evident that effective interventions to support self‐management of lower limb wounds are needed.

This scoping review aimed to explore what interventions currently exist and to answer the following questions:

- How is self‐management in lower limb wounds defined?
- What interventions exist to aid self‐management in lower limb wounds?
- How are self‐management interventions evaluated and what outcome measures are used?
- What is the impact of self‐management interventions on outcomes measured?
- What is the feasibility and acceptability of developing and delivering these interventions?

## 2 MATERIALS AND METHODS

This review was informed by Arksey and O'Malley's framework,^14^ which outlines the five stages required for a comprehensive scoping review; identifying the research question, identifying relevant studies, study selection, charting the data and collating, and summarising and reporting the results. The following eligibility criteria were applied.

### 2.1 Inclusion criteria

- Studies evaluating a supportive self‐management intervention for adults aged over 18 with a diagnosed lower limb wound/ulcer. Studies of mixed populations were included if the majority of patients (over 50%) presented with a lower limb wound not in the exclusion criteria.
- Studied including patients irrespective of previous surgical intervention to treat the underlying cause of ulceration.
- Quantitative and mixed methods studies including randomised controlled trials, cohort studies, case‐control studies, qualitative studies.

### 2.2 Exclusion

Studies related to self‐management interventions in people with any of the following ulcer‐causing conditions (due to the difference in treatment guidelines):

- Sickle cell diagnosis.
- Diabetic ulceration.
- Ulcers caused by solely trauma with no other underlying causality.
- Ulcers caused by vasculitis.
- Pressure ulcers.

#### 2.2.1 Search strategy

The search strategy included papers published between the years 2000 and 2021. Previously published Cochrane reviews whose aim was the evaluation of healthcare interventions, self‐management, and lower limb wounds were used to inform the development of the search strategy.^15^, ^16^, ^17^, ^18^ A full list of search terms can be found in Appendix 1.

An electronic search was carried out using the following databases; CINAHL, EMBASE, Medline, and Cochrane. Additionally, the bibliographies of all studies eligible for inclusion identified by the search strategy were searched for further relevant studies.

#### 2.2.2 Study selection and data extraction

**FIGURE 1:**
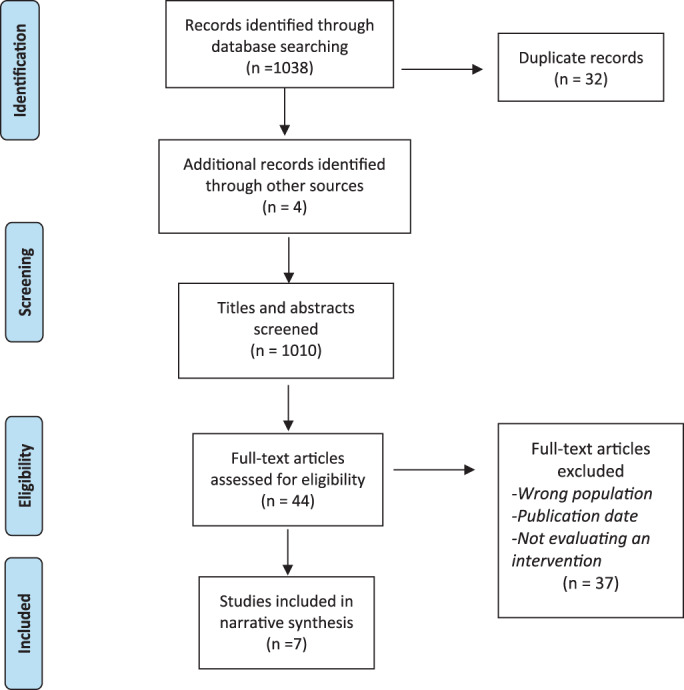
PRISMA diagram

Three authors were involved in reviewing publications for inclusion, LBS, SR & SL. Authors LBS and SR carried out the initial abstract review. Authors to LBS, SR & SL carried out the full‐text reviews and all papers at this stage were reviewed by a minimum of two authors. Where disagreement occurred the third named author was consulted. A breakdown of the study selection process is detailed in Figure 1. Data extraction was informed by the TiDiER framework (template for intervention description and replication)^19^ and covered the 12 required items outlined in Table 1. Data extraction was performed independently by two named authors for each paper and then compared (LBS, SR, SL).

**TABLE 1:** TiDiER framework

	TiDiER framework
Item one	Name of intervention
Item two	Rationale and theory for intervention
Item three	Physical or informational materials required for the intervention
Item four	Intervention procedures or activities
Item five	Provider of intervention (e.g, nurse/doctor/researcher)
Item six	Modes of delivery (e.g, face to face/telephone consultations)
Item seven	Location of intervention
Item eight	Delivery and time periods of intervention
Item nine	Tailoring of intervention (e.g, personalised, titrated or adapted)
Item ten	Intervention modifications
Item elven	Intervention evaluation
Item twelve	Intervention adherence

## 3 RESULTS

Of 1038 articles a total of seven met the inclusion criteria for this review (see Figure 1) and the studies included consisted of three randomised controlled trials, three cohort studies, and one comparative study. Although the search did uncover qualitative studies of self‐management, none specifically looked at an intervention and were therefore excluded.^11^, ^20^, ^21^, ^22^, ^23^ Studies were undertaken in various countries within Europe, the USA, Australia, and Asia. The seven articles represent a population of 735 people living with lower limb wounds with a mean age of 67.6 (±4.05). Sample sizes ranged from 24 participants to 351 and included slightly more women than males (56.19% vs 43.81%).

### 3.1 Definitions of self‐management

Each article described patient activities associated with their supportive self‐management intervention, but none provided a definition of self‐management. Activities varied across studies and included: exercise, wound management, skin care, the application of compression therapy, adherence to hosiery, and the adoption of optimal nutritional habits. Across the included studies four utilised the term ‘self‐management’^24^, ^25^, ^26^, ^27^ and one used “self‐care”.^28^ The distinction between self‐management activities and normal healthy lifestyle behaviours was not always clarified. This is particularly evident in the study published by Miller et al^26^ where they describe self‐management tasks as “healthy lifestyle behaviours associated with delaying ulcer recurrence”.

### 3.2 Interventions for self‐management

Supportive self‐management interventions included a variety of components such as patient education, physical activity, wound management training, skin management, and nutrition. None of the studies included interventions addressing the mental health aspects of self‐management. All interventions focused on participants, excluding family and caregivers. Six of the seven interventions were overseen by nursing staff,^24^, ^25^, ^26^, ^28^, ^29^, ^30^ and five were delivered in a healthcare setting.^24^, ^26^, ^28^, ^29^, ^30^ The remaining two interventions included initial visits to a healthcare setting with further management via telephone or other remote technology such as phone applications.^25^, ^27^ Two interventions utilised goal setting,^24^, ^25^ and two included the use of a tracking device.^25^, ^27^ Full characteristics of included studies are detailed in Table 2, which outlines the key aspects of the TiDiER framework.

**TABLE 2:** Summary of study characteristics

Article	Origin	Study Design	Study population	Name of intervention	Rationale and theory	Definition of self‐management in the context of the study	Intervention	Provider of intervention	Modes of delivery	Location	Delivery /timepoints	Tailoring of intervention
O'Brien et al^25^	Australia	RCT	*n* = 62 Mean Age: Control 71·7 / Intervention 71·3 Sex: Female 30 Male 32 Wound Type: Venous Ulceration Median Wound Duration: Control 14 weeks / Intervention 16 weeks	Keep it up & Taking care of your legs	Social Cognitive Theory	Home‐based exercise programme facilitated by telephone management	Home‐based exercise programme supported by goal setting and regular contact with nursing staff	Research nurse	‐Initially a Face to face appointment ‐Follow up Telephone calls ‐Supplementary written guidance *(booklet/worksheets/pedometer)*	Home‐based	12‐week program Including telephone calls (Weeks −1,2,4,6,8,12)	Titrated ‐ Progressive resistance exercise program Only graduated to the next level upon successful completion of the current level for at least 3 days
Kelechi et al^27^	USA	RCT	*n* = 24 Mean Age: Control 60.7 / Intervention 69.1 Sex: Female 10 Male 14 Wound Type: Venous Ulceration Mean Wound Duration: Control 35 Months / Intervention 27.1 Months	FOOTFIT & FOOTFIT+	Information not provided	Self‐managed exercise intervention to strengthen the lower extremities of minimally ambulatory patients with VLUs	Exercise programme supported by an app that allows patient‐provider communication feature	Study coordinator (Professional status not reported)	Initial Face to face appointment *FOOTFIT‐*Supplementary Bluetooth enabled triaxial accelerometer and app (BEAT). *FOOTFIT+* Supplementary Mobile application also including an additional patient‐provider communication feature.	Home Based	Baseline + Week 6	Titrated‐progressive exercises
Heinen et al^24^	Netherlands	RCT	*n* = 184 Mean Age: Control 67 / Intervention 65 Sex: Female 74 Male 110 Wound Type: Venous or mixed aetiology ulcer Mean Wound Duration: Control 7.3 Months / Intervention 7.0 Months	Lively Legs	Social Cognitive Theory	Physical activity behaviours and adherence to compression therapy	Nurse‐led self‐management counselling programme	Nurses – Trained as health counsellors	Face to Face	Clinic Based	Baseline Week two Week four Six months (3 additional appointments could take place should a patient require extra assistance)	Personalised‐ goals were set tailored to the individual patient needs and opportunities.
Mościcka et al^30^	Poland	Retrospective cohort analysis.	*n* = 351 Mean Age: 64.5 Years Sex: Female 232 Male 119 Wound Type: Venous Ulceration Median Wound Duration: 26.63 ± 20.66 weeks	N/A	N/A	The application of compression therapy	Educational activities focusing on disease management in addition to regular check‐ups with doctors and nurses	Nurse	Face to face	Clinic Based	12‐weeks program ‐one session per week for the first month ‐one session every 2 weeks for the second month *(If ulcer unhealed education continued at all follow up appointments)*	Standardised Program
Miller et al^26^	Australia	Prospective single sample cohort study	*n* = 49 Mean Age: 76.1 Sex: Female 37 Male 12 Wound Type: leg ulcer (not defined) Average Wound Duration: 26.63 Months	Leg Ulcer Prevention Program	N/A	Healthy lifestyle behaviours including: Leg Ulcer Treatment Activity and Exercise Skin Care Nutrition and Hydration Compression stockings for recurrence prevention	Standardised e‐Learning client education package delivering best practice recommendations for venous leg ulcer management in addition to nurse‐led education.	Nurse (provided with training prior to being involved)	Face to face & e‐Learning Supplementary summary sheet and worksheet activity to reinforce the learning.	Clinic Based	6‐week program ‐One session per week for 6 weeks	Standardised Program
Kelechi et al^29^	USA	Comparative study	*n* = 24 Mean Age: Control 65.8 / Intervention 64.8 Sex: Female 8 Male 13 Wound Type: Venous, arterial, and neuropathic leg or foot ulcers (91% venous ulcers) Age of ulcer in months: 0–6: 23% 7–12: 9% 13–18: 4% 19–24: 13% >25: 38%	MECALF	Developed based on previous work evaluating Motivational enhancement	adopt physical activity habits that improve leg condition, reduce pain, and enhance overall health.	Exercise program supported by motivational enhancement and supplementary learning materials.	Nurses (provided with training prior to being involved)	Face to face Supplementary written guidance (brochure)	Clinic Based	6‐week program −10 minutes of the intervention at each wound care visit	Personalised program
Suehiro et al^28^	Japan	Retrospective cohort study	*n* = 41 Mean Age: 68 Sex: Female 22 male 14 Wound Type: Venous Ulceration Median Wound Duration: 0.6 years	N/A	N/A	self‐care‐based strategy involving “no‐ intentional‐stretch” bandaging technique	Education and assessment of compression management application.	Clinicians (Type of clinicians not stated)	Face to face	Clinic Based	Every 1–2 weeks until compression application skills mastered Once skills gained every 1–3 months	Standardised Program

#### 3.2.1 Intervention procedures or activities

Physical activity was the most common component across six of the seven studies and was included in all four interventions that involved multiple components.^25^, ^27^, ^29^ The interventions aiming to promote regular exercise uptake included the following; stretching, walking, toe and foot taps, dorsiflexion, plantar flexion, lower extremity kickouts, ankle twirls/circles, ankle inversion, eversion and pumping.^24^, ^25^, ^26^, ^27^, ^29^ The patient education components of interventions included the cause of venous insufficiency, the correct application of compression therapy, physical activity, skin maintenance such as the use of a soap substitute, and nutrition^25^, ^26^, ^29^, ^30^, ^31^ Two studies looked particularly at the application of compression therapy.^28^, ^30^ The first evaluated the implementation of a compression therapy self‐application programme due to a shortage of appropriately trained staff. An initial education session was given at routine clinical appointments and patients were seen every 1–2 weeks until staff were satisfied they could manage independently.^28^ The second focused on patients' own subjective self‐assessment of their knowledge regarding compression therapy compared with that of the trained nurses' assessments of the patient's compression therapy skills.^30^ A further two studies also incorporated nurse‐led counselling and goal setting to address patient‐reported barriers and facilitators to treatment adherence.^24^, ^29^

#### 3.2.2 Rationale and theory for intervention

None of the included studies described using a validated development framework such as the Medical Research Council's complex intervention framework^32^ to design their intervention. Two of the seven studies utilised behavioural theory in the development of their self‐management intervention.^24^, ^25^ Both detail the use of Social Cognitive Theory (SCT) and provide a brief explanation of how it was applied in addition to citing previous publications where it is explored in more detail^31^, ^33^ When applying the SCT to develop an intervention aimed at behaviours associated with physical activity and adherence to compression therapy, authors report using intervention mapping methodology however they do not state how this was done and what resources were used to guide this process. Authors report that intervention mapping enabled them to perform in‐depth assessments of the needs of patients and determinants of behaviour in order to select evidence‐based methods and strategies that would be most useful. Although a third study Kelechi et al^29^ did not specify a specific behavioural theory, the authors applied the theory of motivational enhancement^34^ in the development of their intervention.

### 3.3 Evaluation of self‐management interventions

All interventions in the review included either goal setting, additional contact with healthcare providers, tracking devices or some combination of the three. Although all of these components seek to influence patient behaviour, only two studies directly evaluated these approaches from the perspective of patient or staff acceptability.^29^ Studies were much more likely to focus on clinical outcomes such as wound healing or the ability to perform specific exercise movements.

A variety of outcome measures were used in the included studies (see Table 3). Although six out of seven interventions included physical activity, they used a range of outcome measures to assess this. Of the six studies including exercise as part of the intervention, all utilised self‐reported exercise measures. The Tinetti Gait and Balance measure, which assesses physical movements associated with balance and gait, was the only measure used in more than one study.

**TABLE 3:** Instruments used to measure outcomes

Instrument	Article
The Pressure Ulcer Healing Score (PUSH) tool for ulcer healing	O'Brien et al^25^
Yale Physical Activity Survey	O'Brien et al^25^
Functional ability measures‐Tinetti Gait and Balance measure	O'Brien et al^25^/Kelechi et al^29^
Range of Ankle Motion (ROM)	O'Brien et al^25^
Exercise adherence‐Likert scale	O'Brien et al^25^
21‐item Foot and Ankle Ability Measure (FAAM)	O'Brien et al^25^
6‐minute walk test	Kelechi et al^27^
The seven‐day Physical Activity Recall inventory (PAR)	Heinen et al^24^
VAS Score	Mościcka et al^30^
Leg Pain Questionnaire (LPQ)	Kelechi et al^29^
Community Healthy Activities Model for Program for Seniors (CHAMPS)	Kelechi et al^29^
Study specific	Heinen et al^24^/Mościcka et al^30^/Miller et al^26^
15‐item Geriatric Depression Scale	Kelechi et al^27^
Questionnaire for physical activity and exercise (self‐efficacy)	Kelechi et al^29^
Medical Outcomes Survey Short Form‐8questionnaire (SF‐8) ‐ SF Physical Component Summary ‐ SF Mental Component Summary	O'Brien et al^25^
12‐Item Short‐Form Health Survey (SF‐12) ‐ SF Physical Component Summary ‐ SF Mental Component Summary	Kelechi et al^27^

Kelechi et al^29^ utilised the most outcome measures relating to exercise, evaluating pain, strength, range of motion, motivation, and functional physical activity. A subsequent study published by Kelechi et al^27^ measured improvement in the function of participants affected ankle and lower limbs.

One study reported outcomes related to wound healing and these data were collected either fortnightly or monthly for 12 weeks after baseline.^25^ Three studies reported ulcer recurrence rates, with one also detailing number of wound days.^24^, ^28^, ^30^ Additional outcomes included the subjective self‐assessment of participants' knowledge,^30^ adoption of specific behaviours^26^, ^29^ and quality‐of‐life measures.^25^, ^27^

### 3.4 The impact of self‐management interventions on outcomes

#### 3.4.1 Physical activity

The impact of interventions on physical activity varied across studies. O'Brien (2017) found limited difference between the intervention and control group in the majority of outcome measures. However, when evaluating the Yale Physical Activity Survey (YPAS), they reported a significant improvement over time in the intervention group, with overall scores being 30 at baseline and 37 at 12 weeks (Wilks *𝜆* = 0.88 [*p* = 0·01]) for the intervention group and no change for the control group (mean score = 21 at baseline and 21 at 12 weeks). (When scoring the YPAS participants can score a minimum of 0 and a maximum of 143). Findings were similar when looking at Range of Ankle Motion (ROAM), where the intervention group also showed improvement over time with the overall scores being 29 at baseline and 32 at 12 weeks (Wilks *𝜆* = 0·92 [*p* = 0.05]) vs a mean score of 23 at baseline and 24 at 12 weeks in the control group. (*The average score for the male general population is 55 to 66 for ROM)*. Although these results are encouraging, the study required 110 participants to be adequately powered to detect change and only enrolled 62. Similarly, although differences in physical activity and movement were found between the control and intervention group in the studies published by Kelechi et al^27^, ^29^ the majority were not clinically significant. This was also thought to be related to the small sample sizes.

Heinen et al^24^ found that patients in the “lively legs” intervention group were more likely to perform leg exercises. However, there was a smaller difference for patients walking for 30 min as opposed to 10, suggesting that shorter walks may be more suitable for patients. When evaluating behaviour adoption, Miller et al^26^ found there was no significant difference in activity levels after participants had completed the intervention, suggested that the intervention had little impact.

#### 3.4.2 Wound healing and management

Of the articles evaluating interventions aimed at self‐management exercise programmes, the two including wound healing or recurrence as an outcome measure suggest a positive relationship between exercise and healing.^24^, ^25^ Heinen et al^24^ observed faster healing rates in the intervention group with a mean of 1.2 months to healing as opposed to 2.5 in the control group (*p* = <0.01). This benefit was maintained at 18 months, although the difference was smaller and not found to be statistically significant, with 56% of patients in the control arm experiencing a new ulcer compared with 46% in the intervention arm. Similarly, O'Brien et al^25^ found that 77% of those in the exercise intervention group healed at week 12 compared to 53% of those who received usual care. However, unfortunately, when using a chi‐squared test for independence, no significant difference was found between the intervention and control groups (*p* = 0.09). As previously mentioned, due to the small number of participants, the study was under powered to detect an effect.

When evaluating patient self‐application of compression therapy, Suehiro et al^28^ found that the median number of visits to learn the bandaging technique was two. Ulcer healing rates at 6 and 12 months were 67% and 86% respectively. 94% of participants were reported to have adhered to prescribed compression therapy. The authors did not state a time point for assessing adherence however the median number of visits to healing was a median of 5 months and it is implied patients were adherent throughout.

The evaluation carried out by Mościcka et al^30^ reviewed patients' self‐assessment of their own knowledge regarding compression therapy. Authors report that the percentage of patients assessing their compression therapy skills as “very good” was significantly higher in the group with recurring ulcers when compared to participants whose ulcers did not recur. However, nurses rated the skill set and knowledge of this patient group poorly, and noted their incorrect or weak knowledge as a significant risk factor for the recurrence of ulceration. The study also evaluated recurrence post‐intervention, with 12.8% of patients experiencing at least one recurrence during the 5 years of follow‐up. The mean time from successful treatment to recurrence was 27.3 +/−16.1 months, however, it is unclear if recurrence was influenced by self‐management.

#### 3.4.3 Adherence, intervention acceptability, and feasibility

As outlined in table two, the intervention evaluated by Kelechi et al^29^ included an exercise program supported by nurse‐led motivational enhancement. Surveys were used to explore acceptability among nursing staff and found that although they felt the intervention was somewhat feasible to deliver during wound care, time was a limiting factor when patients had shorter appointments. Nurses also believed that modifications needed to be made to the staff training programme, as the 1‐day eight‐hour training felt too compact. Study participants reported finding discussion time with their nurses helpful and explained the resources were easy to follow.

When exploring the adoption of healthy lifestyle behaviours associated with delaying ulcer recurrence, the intervention trialled by Miller et al included an educational programme which aimed to improve patients' knowledge of venous disease, and recommended management strategies. It also included dedicated time and activities allowing patients to practice recommended care. While the percentage of patients performing all five behaviours *(heel raises & squats/elevating legs/using soap substitute/moisturising/physical activity)* varied over time, performance was higher for each behaviour at 26 weeks than before receiving the educational programme. Although activity levels and the use of a soap substitute remained fairly stable across all follow‐up time points *(pre‐education/baseline/13 weeks/26 weeks)*, there was a significant decline in the performance of leg elevation, heel raises and squats over time.

Kelechi et al^29^ also reported data on the adoption of behaviour related to exercise. Unfortunately, although the control group believed exercise was a good idea, they did not see it as a priority while they were “waiting for their ulcer to heal”. In comparison, the intervention group displayed a better understanding of the importance of exercise and its effect on limb health. This seems to translate to adoption of behaviour, as 83% of the intervention group reported exercising between six and eight weeks as opposed to 44% of patients in the control group.

#### 3.4.4 Quality of life (QOL)

Of the two studies reporting QOL outcomes, neither reported differences between the intervention and control group.^25^, ^27^ Kelechi et al^27^ did report that in both groups the overall physical and mental health of patients with a lower limb wound was lower than the average reported score across the literature for people of their age.

## 4 DISCUSSION

This scoping review aimed to explore the self‐management of lower limb wounds and the interventions available to aid patients. Unfortunately, no studies actually defined the concept of self‐management in this context. While the studies provide some insight as to what activities might be classed as self‐management, there is limited consensus on what a self‐management intervention for lower limb wounds should include. Almost all interventions described as self‐management interventions included an exercise component, and education programme included learning on nutrition and hydration. However, the degree to which self‐management activities go beyond what would be considered a normal healthy lifestyle behaviour is unclear.

The majority of interventions in this review include more than one component and therefore fulfil the Medical Research Council definition of a complex healthcare intervention, which includes several interacting components.^32^ However, no studies cited the MRC guidance, and few incorporated its key principles. For example, the guidance emphasises the importance of applying theory to the development of interventions, but only three of the included studies referred to previous theory application. It is also interesting that although many interventions included goal setting, only Kelechi et al^29^ utilised a validated self‐efficacy scale *(Questionnaire for physical activity and exercise)*. Furthermore, multi‐component interventions require a comprehensive evaluation of user acceptance.^32^ For this patient group, acceptability and uptake are particularly important, as studies have found that dissatisfaction and mistrust of healthcare services may be a reason for choosing to self‐manage.^11^, ^21^, ^23^ These feelings are likely to influence engagement with healthcare services and could have an impact on the feasibility of interventions. Although many interventions incorporated the delivery of patient education and additional contact with healthcare providers, only Kelechi et al^29^ explored the feasibility of implementation alongside the usual standard of care. The studies, therefore, provide little insight into the acceptability and potential uptake of interventions if they were to be translated into everyday practice.

The qualitative literature describing patients' experiences provides additional insight into factors that may influence self‐management. Several studies describe caregivers as providing significant support, either by carrying out wound dressings or providing motivation for healthy lifestyle behaviours,^20^, ^21^ although it is also the case that family and caregivers can limit care.^22^ Unfortunately, none of the studies in this review incorporated or evaluated the contribution of caregivers. Moreover, the significant quality of life and psychological impact associated with self‐management was only included as a secondary outcome in two studies.

The presented studies demonstrate that there is scope for patients to become more involved in the care of a lower limb wound, but the theoretical and empirical basis for self‐management interventions is weak. There is insufficient evidence regarding the optimal components of self‐management interventions, and for the degree to which patients engage with these. Few studies have evaluated patient or staff acceptability. In an era of healthcare in which we must promote greater self‐management, there is a need for more detailed work in this area, informed by theory, behavioural science, and intervention development frameworks.

### 4.1 Limitations

Although this review was carried out systematically and followed a pre‐determined search strategy a formal protocol was not published and therefore it was not peer reviewed. In addition, although several steps were taken to ensure a robust search strategy there is always the possibility that some relevant studied were not identified. When conducting the analysis the main limitation of this review was the small number of studies identified, making it difficult to fully answer the research questions. Due to the heterogeneity of included studies, it also made it too difficult to carry out a quality appraisal. Lastly, due to the covid‐19 pandemic, the pathway of care for this patient group has changed significantly. This presents a stronger argument for further primary research in this area.

## FUNDING INFORMATION

L Bolton Saghdaoui, S Lampridou, and S Racaru are funded by pre‐doctoral fellowship awarded from Imperial Health Charity/National Institute for Health Research (NIHR) Imperial BRC and NIHR/HEE. M Wells is supported by the NIHR Imperial Biomedical Research Centre (BRC). The views expressed are those of the authors and not those of IHC, NIHR or the Department of Health and Social Care.

## CONFLICT OF INTEREST

No conflict of interest to declare.

## Data Availability

The data that support the findings of this study are available from the corresponding author upon reasonable request

## APPENDIX 1 Search terms

**Wounds**

Lower limb / leg ADJ2 wound / ulcer* / wound.

**Self‐care**

self‐manag* / self‐car*/ self‐efficac* / self‐administration.

&

self or oneself or herself or himself or themsel* adj3 administ* or aid or assert* or assess* or assur* or care* or caring or control* or concept or depend* or determin* or diagnos* or direct* or dosag* or dose* or dosing or evaluat* or efficac* or efficienc* or exam* or govern* or guide* or guidance or help* or maintain* or manag* or medicat* or monitor* or motivat* or regulat* or refer* or rely* or relian* or serve* or serving or service* or subsist* or sufficien* or sustain* or support* or triag.

&

Independent manag / Independent car* / Independent report / Independent monitor* / diagnostic self‐evaluation / self‐help groups.

**Intervention**

Educat / train* or instruct* / patient‐education or “patient education” / “patient cent*” or patient‐cent*/ patient‐focus* or “patient focus*” / patient‐education or “patient education” / “management plan” or management‐plan / management* NEAR1 program* / behaviour* or behaviour* / Behaviour Therapy / Intervention*/ Cognitive Therapy / Health Program / Health Education / Learning / Patient Participation/ EMPOWERMENT/ social support / support system* / social adj support or network / Health Promotion / holistic or wholisti.
